# The fodder grass resources for ruminants: A indigenous treasure of local communities of Thal desert Punjab, Pakistan

**DOI:** 10.1371/journal.pone.0224061

**Published:** 2020-03-05

**Authors:** Humaira Shaheen, Rahmatuallah Qureshi, Mirza Faisal Qaseem, Piero Bruschi

**Affiliations:** 1 COMSATS University Islamabad, Pakistan; 2 Key Laboratory of Plant Resources Conservation & Sustainable Utilization/ Key Laboratory of Digital Botanical Garden of Guangdong Province, South China Botanical Garden, Chinese Academy of Sciences, Guangzhou, China; 3 Pir Mehr Ali Shah Arid Agriculture University Rawalpindi, Pakistan; 4 State Key Laboratory for Conservation and Utilization of Subtropical Agro-bioresources, Guangzhou, China; 5 Guangdong Key Laboratory for Innovative Development and Utilization of Forest Plant Germplasm, College of Forestry and Landscape Architectures, South China Agricultural University. Guangzhou, China; 6 Department of Agricultural, Environmental, Food and Forestry Science (DAGRI) University of Florence, Italy; Universidad Mayor de San Andrés, PLURINATIONAL STATE OF BOLIVIA

## Abstract

Indigenous people have been using local grasses for rearing their animals for centuries. The present study is the first record of traditional knowledge of grasses and livestock feeding system from the Thal desert in Pakistan. A snowball sampling method was used to identify key participants. Information was collected from the respondents from six districts of Thal Desert through semi-structural questionnaire and site visits. The data was analyzed through Smith’s salience index and Composite Salience using ANTHROPAC package in R software. On the whole 61 grasses were recorded from the study area: most of them belong to the Poaceae family (52 species). Based on palatability grasses were categorized into three major groups i.e. (A) High priority, (B) Medium priority and (C) Low priority. Species in Group A, abundantly present in the study area represent a source of highly palatable forage for all ruminants. 232 (141M +91W) local participants were interviewed. Participants were grouped into three major age categories: 20–35 (48 participants), 36–50 (116 participants) and 51–67 years old (68 participants). ANTHROPAC frequency analysis confirmed the Smith’s salience index and Composite Salience; *Cynodon dactylon* was the favorite species (6.46 SI, 0.6460 CS) followed by *Cymbopogon jwarancusa* (5.133 SI, 0.5133 CS) and *Sorghum* sp. was the third most salient species (5.121 SI, 0.5121 CS). Grasses were mostly available during the months of August and October and had also ethnoveterinary importance. This document about the traditional feeding of livestock in Thal Desert can underline the importance of conserving a traditional knowledge, which was poorly documented before.

## Background

In rural areas of Pakistan, agro-pastoral activities play a crucial role in the development of the local economy, accounting for more than half of the total agricultural income and 10.6% of the national GDP [1]. These activities are particularly important in the economy of the country’s desert regions where land cultivation is difficult and livestock husbandry is the main and often unique survival strategy and income source for the local communities. Moreover, milk and meat production may counteract the impact of climatic unpredictability on fluctuations in food availability, especially in areas facing frequent crop shortages. According to data reported by Farooq et al. [2], in Pakistan 8.1% of buffaloes, 13.5% of cattle, 15.3% of sheep and 14.4% of goats are raised in desert districts. However, husbandry in these areas is often an uncertain and low-paid activity; shortage of fodder as a result of severe climatic conditions, high rate of diseases, limited availability of veterinary services and poor access to animal vaccination are important constraints limiting the local livestock productivity [2]. The sustainable production of livestock under harsh climatic conditions needs efficient strategies for improving fodder utilization and management [3]. From this perspective, traditional knowledge can be an important source of information on local wild forage resources and on their nutritive properties. Several studies have shown that smallholder farmers in many parts of the world have a deep practical knowledge about the importance and quality of plants used to feed animals. Ethnobotanical investigations on fodder plants have been carried out in Africa [4–6], Brazil [7], India [8, 9] and China [10–12]. Many studies throughout the world highlight the diverse and abundant use of grasses and sedges as fodder; grasses and sedges are generally reported to be palatable and highly productive resources and to have high forage potential especially in arid and semiarid areas [12, 13].

Previous studies have shown that Thal is rich in grasses and sedges [14]; most of the grasses are used by local population as fodder [10, 13, 15]. However, no detailed study carried out to analyze utilization and selection strategies of these plants by shepherds and farmers living in this zone. Extensive areas in the Thal have been overgrazed and are now strongly threatened by desertification [16, 17]. Understanding the relative importance and preference of different species is crucial for a sustainable management of the local forage resources and can help animal husbandry technicians to optimize the selection of useful fodder species and to improve the livestock system efficiency. Moreover, recording this knowledge would be a much faster and cheaper method for learning about palatability and nutritive value of these plants.

The major aims of this study were: (1) To document traditional knowledge about the use of grasses and sedges as fodder in Thal and to assess similarities and differences with the studies previously conducted in the same [15] and in neighboring areas [11, 12]. (2) To evaluate the impact of socioeconomic factors on the local ethnobotanical knowledge. (3) To rank, by order of preference, the different species used in the animal diet. (4) To quantify the influence of seasonal variation on the availability of these plants as animal feed.

## Materials and methods

### Description of the study area

**Fig 1:**
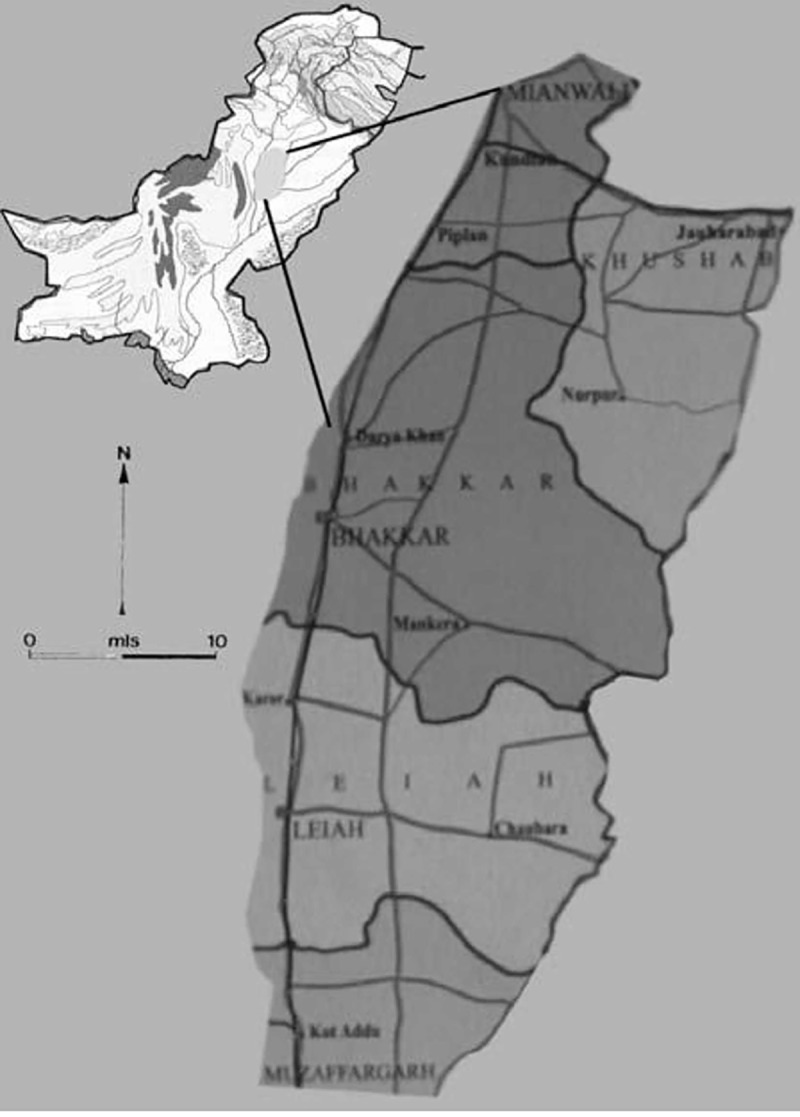
Map of the Thal desert area.

The Thal desert is located between 31° 10’ N and 71° 30’ E in the Punjab province, Pakistan (Fig 1). It is a subtropical sandy desert lying between the Indus River flood plains in the west and Jhelum and Chenab River flood plains in the east. About 50% of the Thal is under arid to hyper-arid climatic conditions (mean annual rainfall less than 200 mm) and the remaining half is characterized by semiarid climatic conditions (annual mean rainfall between 200 and 500 mm). Most of rainfall occurs between June and August. Average temperatures range between 3–8°C in winter and 32–40°C in summer. Wind erosion is a serious problem leading to the loss of topsoil and organic matter and damage to crop plants.

This region is divided into six districts viz. Bhakkar, Khushab, Mianwali, Jhang, Layyah, and Muzaffargarh.

In Thal desert livestock is considered as a secure source of income for small farmers and the landless poor. According to Husain [18] the average herd size is 17 standard animal units. Livestock herds consist of animals of different age and sex; on average each farm has 22.8 goats, 16.7 sheep, 7 cattle, 2.51 buffaloes, 0.88 camels, 0.21 donkeys and 0.05 mules. Detailed information on grazing and stall feeding practiced in the area is given in Faraz et al.[19].

### Ethnobotanical survey

**Fig 2:**
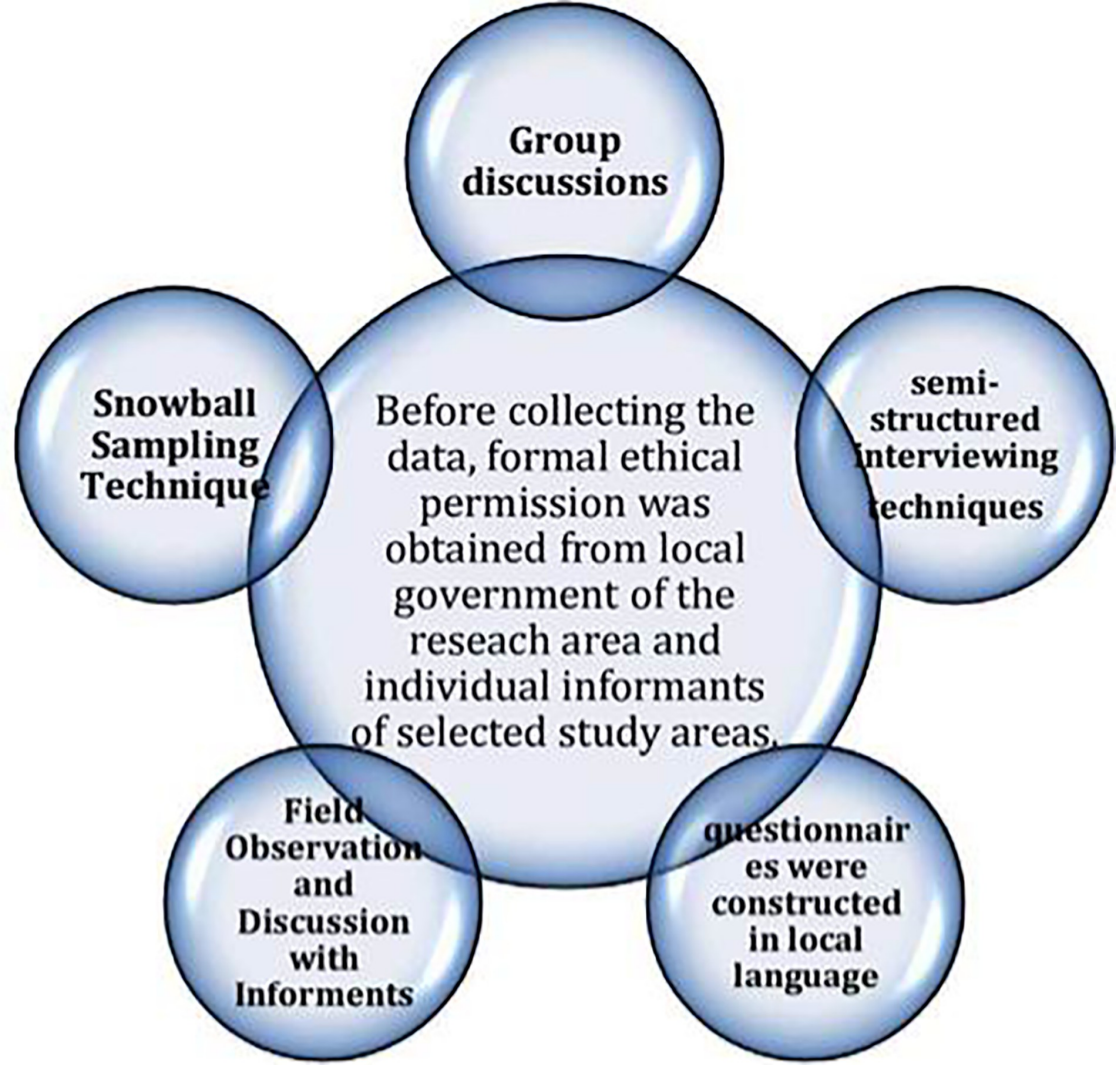
Ethnobotanical survey and data collection.

The ethics committee/IRB of Pir Mehr Ali Shah Arid Agriculture University, Rawalpindi approved this study. Formal ethical consent was also obtained from all participants before the research started. Data were collected for two consecutive years (from March 2016 to March 2018), twice a year from each of the Thal desert six districts. Participants were selected by snowball-sampling technique [20] among village leaders, shepherds and both farm and domestic livestock caretakers. Interviews were carried out complying with the ethics guidelines commonly followed in ethnobotanical studies [21, 22]. Information was gathered by using different approaches i.e. group discussions with participants, individual semi-structured questionnaires and participant observation (Fig 2) [23, 24]. The questionnaires were drafted in the local language (*Seriki* and *Punjabi*) and included the following major questions: (i) Which grasses/sedges are used as fodder? (ii) Which grasses/sedges are preferred as feed for cattle, sheep, camels, buffaloes, and goats? (iii) What is the palatability of the different used plants? (iv) Which plant part do animals consume? (v) What are the feeding habits of different animals? (vi) Which livestock feeding system does local people adopt: free grazing or cut and carry? (vii) Do the listed fodder plants have any ethnoveterinary use? (viii) What are their other indigenous uses?

In the second stage of the field research we used direct observation of livestock grazing habits to evaluate the palatability of different plants, animal preferences and the growth stages of plants at the time of grazing.

### Collection and identification of plants

**Fig 3:**
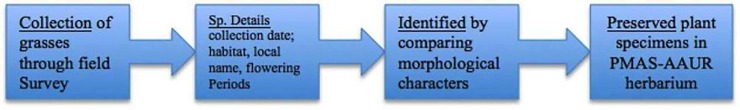
Different steps in the collection and identification of grasses.

Plant collection was performed with the help of local participants during the field survey. Identification of the gathered species was carried out by the herbarium specialist Dr. Mushtaq Ahmed from Quaid-i-Azam University, Islamabad and by the taxonomist Dr. Humaira Shaheen (Fig 3). Botanical nomenclature of species and families complies with online Flora of Pakistan (http://www.efloras.org/flora_page.aspx?flora_id=5) [24] and the herbarium specimens were kept in the Botany Department of Pir Mehr Ali Shah University of Arid Agriculture.

### Data analysis

The most common method to measure relative abundance was visual assessment and observation of ethnobotanically important grasses in the study area [12]. Total study area was almost 20,000 square kilometers. We randomly divided each district into 45–50 plots and plot size was (10X10m = 100m^2^). Results were constructed by percentage of relative abundance through the following formula:

$$RA=\frac{Total\;percentage\;cover\;of\;species\;over\;all\;plots}{Number\;of\;plots\;estimated}\times 100$$

Based on the abundance value, grasses were categorized into the following groups i.e. abundant, common, frequent, occasional and rare (Table 1).

**Table 1:** Relative abundance categories and coverage in the study area.

**Abundance scale**	**Abundance categories**	**Coverage of Grasses**
	Rare (R)	<7%
1	Occasional (O)	7–10%
2	Frequent (F)	10–25%
3	Common (C)	25–55%
4	Abundant (A)	55–100%

Relative frequency of citation (RFC) was calculated to sort listed plants by priority order, using the following formula [12, 24–26]:

$$RFC=\frac{fc}{n}$$

Where *fc* is the number of participants that mentioned the fodder use of the species and “n” is the total number of participants included in the study.

Pairwise comparison (PWC) was also used to determine the priority order of the listed species [12, 27]. Ten participants (5 key participants and 5 randomly selected) were chosen for the PWC. The participants were asked, one at a time, to select their preferred fodder plants from all possible pairs of species. Each species got a score of 1 if the participants selected it. Adding the scores and ranking them to obtained the final score.

Smith’s salience index and Composite Salience [28] were used to evaluate species saliency by weighing the average of the inverse rank of a species across multiple free-lists where each list was weighed by the number of species in the list. ANTHROPAC [28] was used to generate Smith’s salience indexes.

Pairwise ranking or comparison was used to evaluate the degree of preference or levels of importance. The values for use reports across the selected species were summed up and ranked. Ten participants (six key and four randomly taken participants) in the study area ranked grasses according to their use e.g. 1st, 2nd, 3rd, 4th and 5^th^ respectively. Ranking can be used for evaluating the degree of preference or level of importance of selected plants [28–30].

Respondent Consensus Factor (Fic): The Respondent consensus factor was derived in order to seek the importance of species used as fodder, Forage, Mixed feed and veterinary uses [31].

$$Fic=\frac{Nur-Nt}{Nur-1}$$

Where Nur is the number of use-reports in each disease category; Nt is number of species used.

### Socioeconomic factors

**Fig 4:**
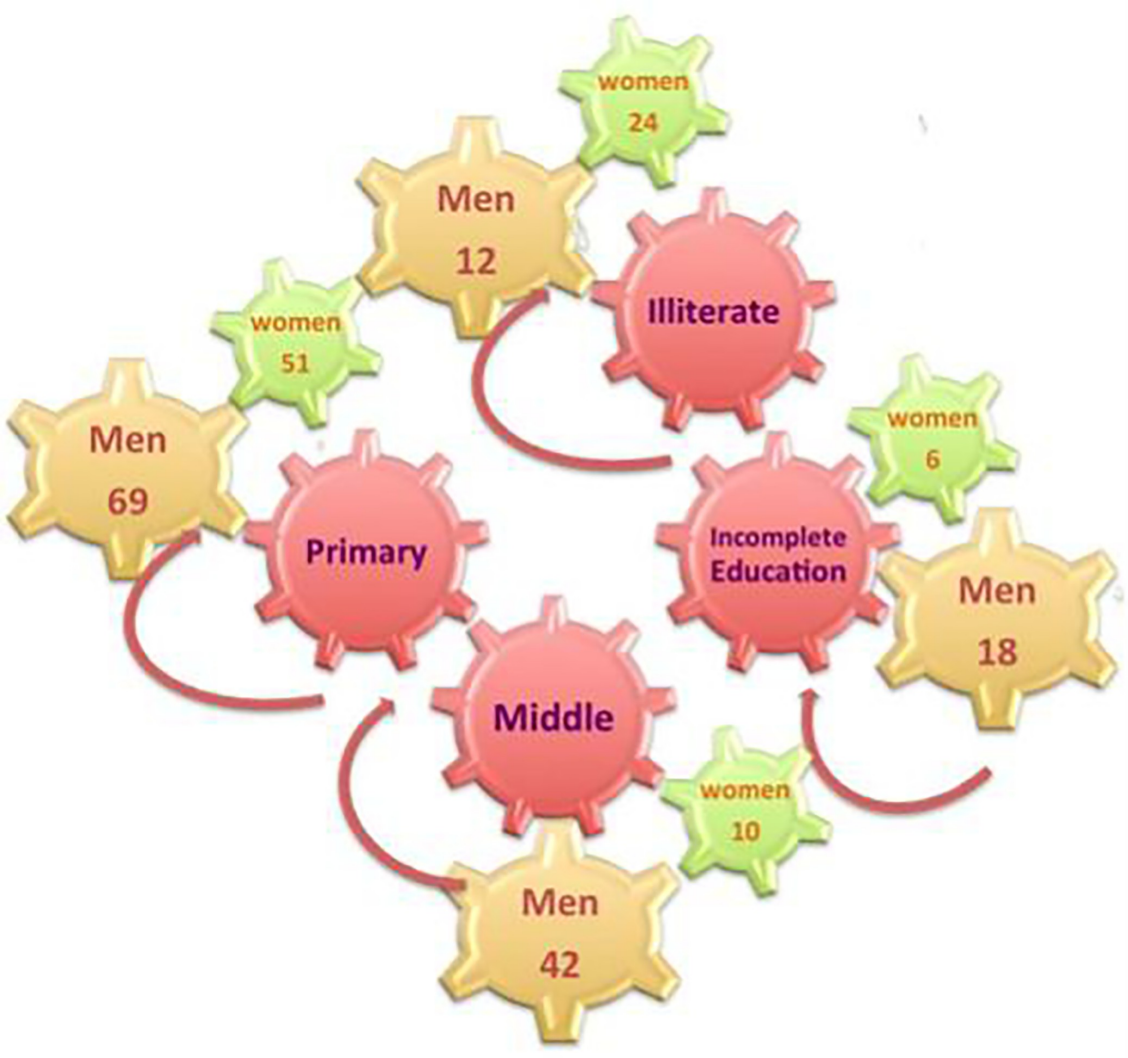
Education levels of participants.

In total, 232 local participants were interviewed (Table 2); 141 were men and 91 were women. A smaller number of female participants were expected and this can be partially explained with the local cultural restrictions preventing women from working outside their homes or farms. Participants were grouped into three major age categories: 20–35 (48 participants), 36–50 (116 participants) and 51–67 years old (68 participants). With regard to the profession, 34% (36 women and 44 men) were shepherds, 26% (27 women and 33 men) were farmed livestock caretakers and 40% (28 women and 64 men) domestic livestock caretakers. Thirty-six (16%) of the interviewed people were illiterate, 24 (10%) never completed their primary education, 120 (52%) completed 5 years of primary school and 52 (22%) participants had middle education level (Fig 4) [24].

**Table 2:** Demography of participants of the study area.

Type of Respondents	Young aged	Middle aged	Seniors aged	Total
	20–35	36–50	51–67	
Local Shepherds (F)	8	19	9	36
Local Shepherds (M)	11	20	13	44
Farmed Ruminant care takers (F)	5	17	5	27
Farmed Ruminant care takers (M)	11	16	6	33
Domestic Ruminant care takers (F)	7	12	9	28
Domestic Ruminant care takers (M)	6	32	26	64
Total Respondents	48	116	68	232

**Key:** Local Shepherds (who take care cattle in the field for free grazing), Farmed Ruminant caretakers (who take care cattle in the livestock forms), Domestic Ruminant caretakers (who take care cattle in their home).

## Results and discussion

### Use of fodder species

The participants reported the use of 61 plant species that were distributed into 40 genera and 3 botanical families. The most represented genus was *Cyperus* with 5 species, followed by *Cenchrus* and *Eragrostis* with 4 species each. Most species belonged to Poaceae family (51 species; 84% of the reported plants) while 8 species (13%) were categorized into Cyperaceae family. Typhaceae were represented by only one species: *Typha elephantina*. Fifty-five species (92% of the reported species) were classified as native and 5 (8%) as exotic. The following exotic species were reported by participants: *Chloris gayana*, *Imperata cylindrica*, *Paspalum dilatatum*, *Sorghum bicolor* and *Vetiveria zizanioides*. These results seem to reflect composition and distribution patterns of the local flora. In a floristic checklist of Thal desert, Shaheen et al. [14] observed that Poaceae was the main family with 52 species. Of the 52 Poaceae naturally occurring in the area, 48 (94%) were reported to be used as fodder in our study; 5 were not cited by participants and 4 (*Brachiaria reptans*, *Eragrostis atrovirens*, *E*. *cilianensis*, *Themeda anathera*) were reported in our study but not in the floristic inventory. All the eight Cyperaceae cited were included in the study conducted by Shaheen et al. [14].

Our comparative analysis revealed that 15 species are used as fodder in all the considered studies. We found a mean similarity (Jaccard index) rather high (36.4 ± 6.9) with values ranging from 30.8 (this study vs [11]) to 50.0 ([12] vs [11]). These studies were all conducted in zones lying in the proximity of the study area that share not only similar ecological factors but also the same socioeconomic and cultural history. Nevertheless, our study listed 20 grasses not previously reported in the fodder category for this area. These results provide an important new contribution to the knowledge on wild fodder plants in Pakistan. At the same time, they also show the importance of collecting new ethnobotanical information even in already studied areas.

### Socioeconomic factors

Participants mentioned 8.27 ± 4.49 taxa (range 1–18). Gender (H = 0.373; P > 0.05) and education (H = 5.29; P> 0.05) had no influence on the knowledge of fodder plants. Gender influence on traditional knowledge is controversial [32] and many studies have showed that the statistical strength of this relation depends on the local cultural context and on the categories of use that the researchers focus on. A lack of differentiation between men and women, as observed in this study, could mean that there is not a clear division of labor in the area. A similar finding was observed by Aumeeruddy et al. [32] in Northern Pakistan, where women have a detailed knowledge on characteristics and properties of the different fodder species, suggesting that they fully share with men the responsibility of livestock rearing and forage collection. Khan and Khan [33] observed that most of the women of Cholistan desert have an important role in managing livestock, spending almost 8 to 13 hours a day in this activity. Differently Nunes et al. [7] and Bruschi et al. [6] showed that men prevail in the knowledge about fodder plants. The greater male knowledge found in these two studies may be explained by different gender-based experiences and skills: men spend much of their time moving with their herds while women are more frequently involved in managing food and family care. The age of participants resulted to be statistically significant (H = 9.97; P < 0.05). As also shown in many other ethnobotanical studies [34–36] elderly people seem to retain more traditional knowledge on the use of plants. For young people (25–35 years old), the average number of known fodder plants was 6.65 ± 4.12 while for middle-aged (36–50) and elderly participants (> 50) there was an average number of 8.25 ± 4.13 and 9.42 ± 4.74, respectively. Occupation also strongly affected the number of fodder species reported by participants (H = 14.58; P < 0.01). Domestic livestock caretakers mentioned a higher number of plants (9.50 ± 4.43) followed by farmed livestock caretakers (7.98 ± 4.02) and shepherds (7.10 ± 4.60). Domestic livestock caretakers spend much time with cattle and have a better knowledge about the animals’ favorite foods.

### Pairwise ranking of wild palatable plants

*Cymbopogon jwarancusa* subsp. *jwarancusa* with 1^st^ rank was the most preferred among all selected grass species, followed by *Cynodon dactylon*, *Cenchrus ciliaris*, *Typha elephantina* and *Cyperus alopecuroides* that had 2^nd^ 3^rd^, 4^th^ and 5^th^ rank respectively. *Pycreus flavidus* received the lowest score, therefore resulting as the less preferred species (Table 3). The most highly ranked species (*Cymbopogon jwarancusa* subsp. *Jwarancusa*, *Cynodon dactylon*, *Cenchrus ciliaris*, *Typha elephantina* and *Cyperus alopecuroides*) are also the most dominant in the area (Shaheen, unpublished data). This finding seems to support the “appearance hypothesis” stating that the most abundant species are better known and mostly used [37]. Plants commonly growing in the area allow local people to have more experience of their properties and consequently have a greater probability of being introduced into the local culture.

**Table 3:** Pair wise ranking of wild palatable plants from all districts of Thal.

**S. No.**	Botanical name	R1	R2	R3	R4	R5	R6	R7	R8	R9	R10	T	R
1	*Cymbopogon jwarancusa subsp*. *jwarancusa* (Jones) Schult.	5	5	5	5	5	5	5	5	5	3	48	1^ST^
2	*Cynodon dactylon* (L.) Pers.	5	4	4	5	4	4	4	5	4	4	43	2^ND^
3	*Cenchrus ciliaris* L.	4	3	4	4	4	5	3	4	4	4	39	3^RD^
4	*Typha elephantina* Roxb.	5	4	5	3	3	5	4	5	3	1	38	4^TH^
5	*Cyperus alopecuroides* Rottb.	4	2	3	3	4	3	5	4	2	3	33	5^TH^
6	*Eragrostis minor* Host	2	2	3	4	4	5	2	2	3	5	32	6^TH^
7	*Sporobolus arabicus* Boiss.	2	3	4	4	3	2	3	2	3	5	31	7^TH^
8	*Brachiaria reptans (L*.*) C*. *A*. *Gardner & C*.*E*.	1	5	4	2	3	1	0	4	5	5	30	8^TH^
9	*Tragus roxburghii* Panigrahi	1	5	4	2	3	1	0	4	5	5	30	9^TH^
10	*Lasiurus sindicus* Henr.	4	2	2	4	5	3	2	2	4	1	29	10^TH^
11	*Aristida funiculate* Trin. & Pupr.	5	4	2	3	1	0	4	5	3	2	29	10^TH^
12	*Cenchrus pennisetiformis* Hochst. & Steud.	1	5	4	2	3	1	0	4	5	4	29	10^TH^
13	*Saccharum spontaneum* L.	2	2	3	4	4	5	2	2	3	2	29	10^TH^
14	*Themeda triandra* Forsk.	5	4	2	3	1	0	4	5	3	2	29	10^TH^
15	*Pycreus flavidus* (Retz.) T. Koyama	2	3	3	2	4	1	3	2	3	5	28	11^TH^

### Correlation used for pairwise comparison

**Fig 5:**
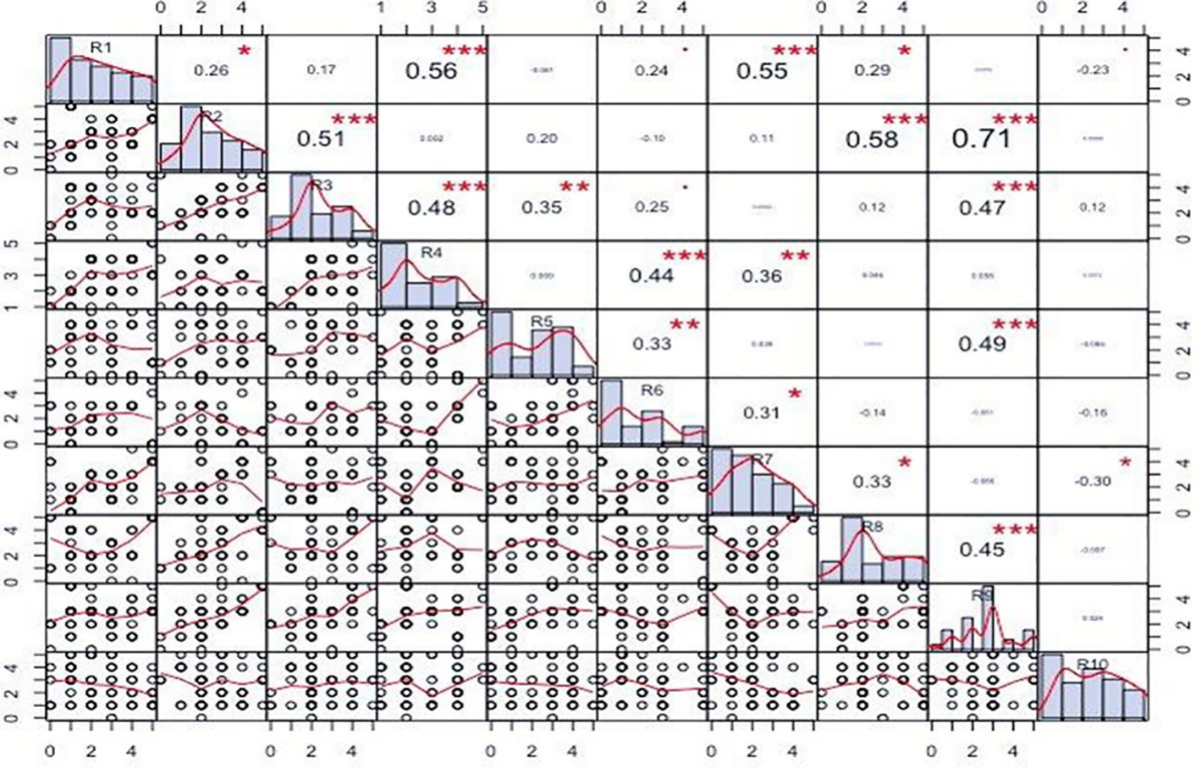
Co-relation used for pairwise comparison of different grasses.

On the basis of RFC value, pairwise comparison was used to correlate fodder grasses and the knowledge of the respondent. Ten out of 232 respondents were chosen on the basis of their profession (ethnoveterinary practitioner) but were potential respondents due to sufficient indigenous knowledge. Based on RFC values knowledge of respondent R1 showed a strong correlation with R4, as R2 (0.56; p<0.001) with R1 and R7 (0.55;p<0.001), R2 had a strong correlation with R3 and R8 (0.48, 0.58; p<0.001) but R2 had the strongest correlation with R9 (0.71; p<0.001). All correlation and the distribution of RFC values are shown in Fig 5. The positive correlation between respondents suggests that respondents report similar information about the plant; for example, R2 and R9 both were ethnoveterinary practitioners more than 50 years old, so they had similar knowledge.

### Availability and prioritizing fodder grasses on the basis of RFC and PWC

**Fig 6:**
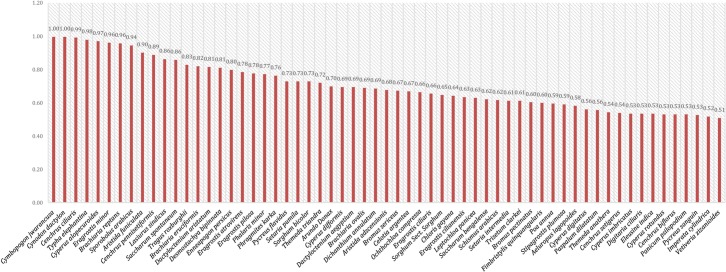
Prioritizing of fodder grasses based on RFC.

**Fig 7:**
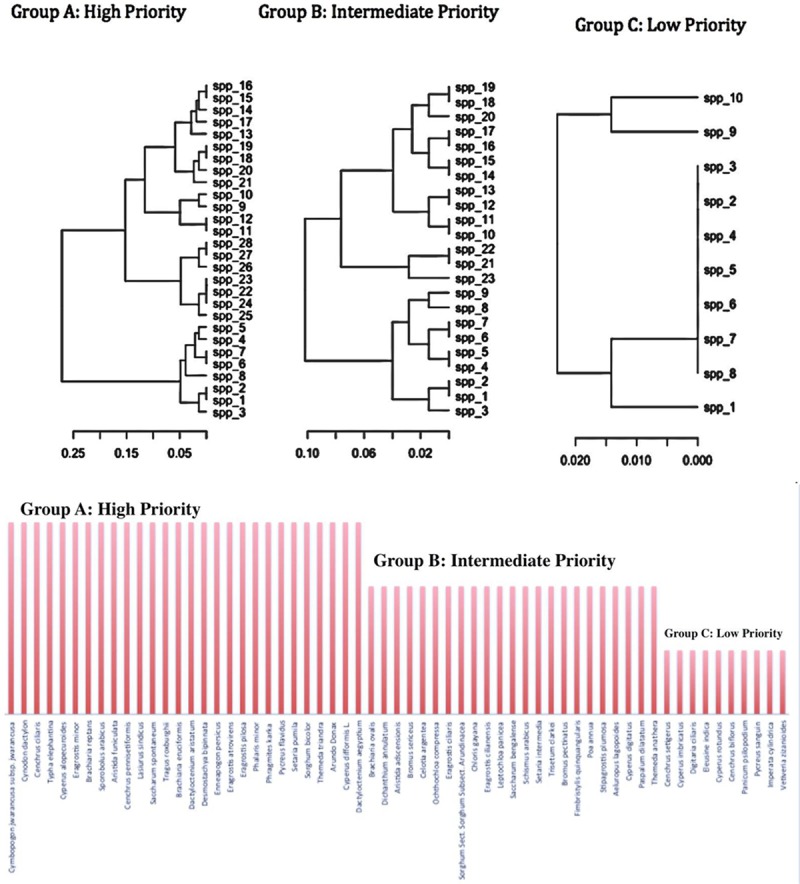
Grouping of ethnobotanically used fodder grasses based on cluster analysis.

RCF values ranged from 1 to 0.51 with a mean value of 0.71. Twenty-five species had RFC values higher than average while the remaining 35 species had RFC value lower than average (Fig 6, Table 4). *Cymbopogon jwarancusa* and *Cynodon dactylon* showed the highest value (1.00) while *Imperata cylindrical* (0.52) and *Vetiveria zizanioides* (0.51) had the lowest. Fic in Table 5 conformed that *Cymbopogon jwarancusa*, *Cynodon dactylon* and *Cenchrus ciliaris* have highly useful as fodder. Based on these RFC values fodder species were classified into three categories of priority: species with higher priority (group A), species with medium priority (group B) and species with low priority (group C). Twenty-eight (45.9%) species were highly preferred by the participants, followed by twenty-three (37.7%) species that had medium priority while ten (16.3%) grass species were the least preferred (Fig 7). Values ranged between 1–0.69 for group A, between 0.69–0.54 for group B and between 0.54–0.51 for group C. Similar results were shown by Harun et al. [12] in their study. These results were confirmed by cluster analysis based on RFC in which the reported species were classified into three major groups compliant with the results of priority ranking analysis. Similar results were found when we performed cluster analysis using PWC data. *Cymbopogon jwarancusa* was the preferred species in both approaches (Table 6).

**Table 4:** List of the collected grasses, ethnobotanical and ethno veterinary data, abundance; focal persons count (FC) and relative frequency citation (RFC) of fodder grasses from the area of Thal desert area, Punjab Pakistan.

**S. No.**	Voucher No	Botanical name	Local name	Palatable	Fodder part	Feeding method	Ethno veterinary	Other uses	Soil ecology	RA	FC(n)	RFC
1	PMAS-AAUR-2013-320	*Cyperus alopecuroides* Rottb.		G, S, B, C, CA	WP	Fo	***NO***	***NO***	***NO***	F	225	0.9698
2	PMAS-AAUR-2013-321	*Cyperus difformis* L.	Bhudde	G, S, B, C	WP	Fo	***NO***	***NO***	***NO***	C	161	0.6940
3	PMAS-AAUR-2013-322	*Cyperus digitatus *Roxb.	Sowe	G, S, B, C	WP	Fo, For	***NO***	Fuel	Soil binder	C	130	0.5603
4	PMAS-AAUR-2013-323	*Cyperus imbricatus* Retz.		G, S, B, C, R	WP	Fo, For	***NO***	***NO***	Soil binder	F	124	0.5345
5	PMAS-AAUR-2013-324	*Cyperus rotundus* L.	Dela	G, S, B, C, R, P	WP	Fo	*YES*	*YES*	Soil binder	A	123	0.5302
6	PMAS-AAUR-2013-325	*Fimbristylis quinquangularis* (Vahl) Kunth	Murrakh	G, S, B, C, R	WP	Fo, For	***NO***	***NO***	Soil binder	O	139	0.5991
7	PMAS-AAUR-2013-325	*Pycreus flavidus* (Retz.) T. Koyama	Sayyar Ghaah	G, S, B, C, R	WP	Fo, For	***NO***	*YES*	Soil binder	O	169	0.7284
8	PMAS-AAUR-2013-327	*Pycreus sanguin* (Vahl) Nees	Ghaa	G, S	AP,JS	Fo	***NO***	*YES*	***NO***	F	122	0.5259
9	PMAS-AAUR-2013-328	*Aeluropus lagopoides (L*.*) Thwaites*	Kalar Ghaah	G, S, C	WP	Fo	***NO***	*YES*	***NO***	A	135	0.5819
10	PMAS-AAUR-2013-329	*Aristida adscensionis* L	*Lamb Ghaas*	G, S	WP	Fo, For	*YES*	***NO***	***NO***	A	157	0.6767
11	PMAS-AAUR-2013-330	*Aristida funiculata* Trin. & Pupr.	*Lamb Ghaas*	G, S, C	WP	Fo, For	***NO***	***NO***	***NO***	A	209	0.9009
12	PMAS-AAUR-2013-331	*Arundo Donax* L.	Narr	G, S, C, B	AP, JS	Fo, For, Mf	*YES*	*YES*	***NO***	A	162	0.6983
13	PMAS-AAUR-2013-333	*Brachiaria eruciformis* (J.E. Smith) Griseb		G, S, B, C	WP	Fo, For	***NO***	***NO***	***NO***	A	190	0.8190
14	PMAS-AAUR-2013-332	*Brachiaria ovalis* Stapf	Ghaa	G, S, C, B	WP	Fo	***NO***	*YES*	***NO***	A	160	0.6897
15	PMAS-AAUR-2013-334	*Brachiaria reptans* (L.) C. A. Gardner & C.E.	Ghaah	G, S, C, B	WP	Fo, For	***NO***	***NO***	***NO***	A	222	0.9569
16	PMAS-AAUR-2013-335	*Bromus pectinatus* Thunb.		G, S	WP	Fo	***NO***	*YES*	***NO***	A	140	0.6034
17	PMAS-AAUR-2013-336	*Bromus sericeus* Drobov		S, G	WP	Fo	***NO***	*YES*	***NO***	A	156	0.6724
18	PMAS-AAUR-2013-337	*Celotia argentea* L.	Ghaah	S, G	WP	Fo	***NO***	***NO***	***NO***		155	0.6681
19	PMAS-AAUR-2013-338	*Cenchrus biflorus* Roxb.	Mohabbat buti/Ludri	S, G	JS	Fo, Mf	*YES*	***NO***	***NO***	A	123	0.5302
20	PMAS-AAUR-2013-339	*Cenchrus ciliaris* L.	Drahman/Dhaman ghaa	G, S, B, C, CA	WP	Fo, For	*YES*	*YES*	***NO***	A	230	0.9914
21	PMAS-AAUR-2013-340	*Cenchrus pennisetiformis* Hochst. & Steud.	Dhamni	S	WP	Fo	***NO***	*YES*	***NO***	A	206	0.8879
22	PMAS-AAUR-2013-341	*Cenchrus setigerus* Vahl	Talra	S, G	WP	Fo	*YES*	*YES*	Soil binder	C	125	0.5388
23	PMAS-AAUR-2013-342	*Chloris gayana* Kunth	Chitta ghaa	S, G	JS	Fo	***NO***	***NO***	***NO***	A	149	0.6422
24	PMAS-AAUR-2013-343	*Cymbopogon jwarancusa subsp*. *jwarancusa* (Jones) Schult.	Khavi	G, S, B, C, CA	AP, JS	Fo, For, Mf	*YES*	*YES*	Soil binder	C	231	0.9957
25	PMAS-AAUR-2013-344	*Cynodon dactylon* (L.) Pers.	Talla	G, S, B, C, CA	AP	Fo, For	*YES*	*YES*	Soil binder	C	231	0.9957
26	PMAS-AAUR-2013-345	*Dactyloctenium aegyptium* (L.) Willd.	Madhana ghaa	S, C	WP	Fo	*YES*	***NO***	Soil binder	C	161	0.6940
27	PMAS-AAUR-2013-346	*Dactyloctenium aristatum*	Madhana	G, S, B	WP	Fo, For	***NO***	***NO***	Soil binder	A	189	0.8147
28	PMAS-AAUR-2013-347	*Desmostachya bipinnata* (L.) Stapf.	Dab Ghaa	G, S, B, C,	WP	Fo, Mf	*YES*	*YES*	Soil binder	A	188	0.8103
29	PMAS-AAUR-2013-348	*Dichanthium annulatum* (Forssk.) Stapf	Murgha ghaa	S, G	WP	Fo	*YES*	*YES*	Soil binder	A	159	0.6853
30	PMAS-AAUR-2013-349	*Digitaria ciliaris* (Retz.) Koel		S, G	AP	Fo, For	***NO***	*YES*	Soil binder	F	124	0.5345
31	PMAS-AAUR-2013-350	*Eleusine indica* (L.) Gaertn.	Gandel ghaa	S	AP	Fo	*YES*	***NO***	Soil binder	C	124	0.5345
32	PMAS-AAUR-2013-351	*Enneapogon persicus *Boiss.		S	AP	Fo	***NO***	***NO***	***NO***	A	185	0.7974
33	PMAS-AAUR-2013-352	*Eragrostis atrovirens* (Desf.) Trin. Ex Steud.	Ghaah	G, S, B, C, CA	WP	Fo, For	***NO***	***NO***	***NO***	A	182	0.7845
34	PMAS-AAUR-2013-353	*Eragrostis cilianensis* (All.) Lut. ex F.T. Hubbard	Ghaa	G, S, B, C, CA	WP	Fo,F or	***NO***	***NO***	***NO***	A	147	0.6336
35	PMAS-AAUR-2013-354	*Eragrostis ciliaris* (L.) R. Br.	Ghaa	S, G	WP	Fo	***NO***	***NO***	***NO***	A	152	0.6552
36	PMAS-AAUR-2013-355	*Eragrostis minor* Host	Ghaa	S, G	WP	Fo	*YES*	***NO***	***NO***	A	223	0.9612
37	PMAS-AAUR-2013-356	*Eragrostis pilosa (Linn*.*) P*. *Beauv*.	* *	G, S, B, C, CA	WP	Fo, For	*YES*	***NO***	***NO***	R	180	0.7759
38	PMAS-AAUR-2013-357	*Imperata cylindrica* (L.) Raeuschel.	Dab Ghaas	S	AP, JS	Fo	***NO***	***NO***	***NO***	O	120	0.5172
39	PMAS-AAUR-2013-358	*Lasiurus sindicus* Henr.	Karera	G, S, B, C, CA	WP	Fo, For	*YES*	***NO***	Soil binder	C	200	0.8621
40	PMAS-AAUR-2013-359	*Leptochloa panicea* (Retz.) Ohwi		S	WP	Fo, Mf	***NO***	*YES*	***NO***	C	146	0.6293
41	PMAS-AAUR-2013-360	*Ochthochloa compressa* (Forssk.) Hilu	Juth Madhaana/Chhimbar/Buchri ghaa	S	AP	Fo	***NO***	***NO***	Soil binder	A	154	0.6638
42	PMAS-AAUR-2013-361	*Panicum psilopodium* Trin.		S	AP	Fo, For, Mf	***NO***	***NO***	Soil binder	C	123	0.5302
43	PMAS-AAUR-2013-362	*Paspalum dilatatum* Poir.	Ghaa	S	WP	Fo	***NO***	***NO***	Soil binder	C	129	0.5560
44	PMAS-AAUR-2013-363	*Phalaris minor* Retz.	Dumbi sitti	G, S, B, C, CA	WP	Fo	***NO***	***NO***	***NO***	F	179	0.7716
45	PMAS-AAUR-2013-364	*Phragmites karka* (Retz.) Trin. ex Steud.	Narr	S, B	L	Fo	***NO***	*YES*	***NO***	C	177	0.7629
46	PMAS-AAUR-2013-365	*Poa annua* L.	Machhar ghaa	G, S, B, C, CA	WP	Fo	***NO***	***NO***	***NO***	C	138	0.5948
47	PMAS-AAUR-2013-366	*Saccharum bengalense* Retz.	Saroo	B, C	L	Fo, For, Mf	*YES*	*YES*	Soil binder	O	144	0.6207
48	PMAS-AAUR-2013-367	*Saccharum spontaneum* L.	Saroo	B, C	L	Fo, For, Mf	*YES*	*YES*	Soil binder	O	199	0.8578
49	PMAS-AAUR-2013-368	*Schismus arabicus* Nees	Ghaa	S	AP	Fo	***NO***	***NO***	Soil binder	A	143	0.6164
50	PMAS-AAUR-2013-369	*Setaria intermedia* Roem. & Schult		S	WP	Fo	***NO***	***NO***	Soil binder	A	142	0.6121
51	PMAS-AAUR-2013-370	*Setaria pumila* (Poir.) Roem. & Schult.		S	WP	Fo	***NO***	***NO***	Soil binder	F	169	0.7284
52	PMAS-AAUR-2013-371	*Sorghum bicolor* (Linn.) Moench.	Milo	G, S, B, C, CA	WP	Fo, For, Mf	*YES*	*YES*	***NO***	A	169	0.7284
53	PMAS-AAUR-2013-372	*Sorghum* Sect. *Sorghum* Subsect. *Arundinacea Moench*.	Milo	G, S, B, C, CA	WP	Fo, Mf	***NO***	*YES*	***NO***	A	150	0.6466
54	PMAS-AAUR-2013-373	*Sporobolus arabicus* Boiss.		G, S, B, C, CA	WP	Fo	***NO***	*YES*	***NO***	A	219	0.9440
55	PMAS-AAUR-2013-374	*Stipagrostis plumosa* (Linn.) Munro ex T.	Chita gah	G, S	WP	Fo	***NO***	***NO***	Soil binder	F	137	0.5905
56	PMAS-AAUR-2013-375	*Themeda anathera*		G, S	WP	Fo, For	***NO***	***NO***	***NO***	F	126	0.5431
57	PMAS-AAUR-2013-376	*Themeda triandra* Forsk.		G, S	WP	Fo, For	***NO***	***NO***	***NO***	R	167	0.7198
58	PMAS-AAUR-2013-377	*Tragus roxburghii* Panigrahi	Ghaa	G, S	WP	Fo	***NO***	*YES*	***NO***	A	192	0.8276
59	PMAS-AAUR-2013-378	*Trisetum clarkei* (Hook.f.) R. R. Stewart		G, S	WP	Fo	***NO***	***NO***	Soil binder	R	142	0.6121
60	PMAS-AAUR-2013-379	Vetiveria zizanioides (Linn.) Nash		G, S	AP	Fo, For	***NO***	***NO***	***NO***	R	118	0.5086
61	PMAS-AAUR-2013-380	*Typha elephantina* Roxb.	Kundar	B, C	L	Fo, For, Mf	***NO***	*YES*	***NO***	F	227	0.9784

Whole plant (WP), Leaves (L), Areal parts (AP), Juvenile stage (JS), Cow (C), Buffalo (B), Goat (G), Sheep (S), Camel (CA), Rabbit (R), Porcupine (P)

Fo, Fodder, For, Forage, Mf, Mix with feed, Goat, RA Relative abundance, A Abundant, C Common, F Frequent, O Occasional, R Rare

**Table 5:** Respondent consensus factor for grasses used by animals.

Use categories	Nt	Nur	Fic	Plants
Fodder	62	7168.9	0.99	*Cyperus alopecuroides*, *Eragrostis minor*, *Aristida funiculata*, *Cynodon dactylon*, *Cenchrus ciliaris*, *Cymbopogon jwarancusa subsp*. *Jwarancusa*, *Typha elephantina*, *Brachiaria reptans*
Forage	27	2299.9	0.98	*Cynodon dactylon*, *Cenchrus ciliaris*,*Aristida funiculata*, *Cymbopogon jwarancusa subsp*. *Jwarancusa*, *Typha elephantina*, *Brachiaria reptans*
Mix with feed	12	501.9	0.97	Cymbopogon jwarancusa subsp. Jwarancusa, *Typha elephantina*, *Saccharum spontaneum*, *Sorghum bicolorDesmostachya bipinnata*
veterinary	18	403	0.95	*Cenchrus ciliaris*, *Cenchrus biflorus*, *Desmostachya bipinnata*, *Cyperus rotundus*, *Cynodon dactylon*, *Digitaria ciliaris*, *Saccharum spontaneum*

**Table 6:** Pairwise comparison (PWC) based on similar RFC vales of fodder grasses.

Fodder grasses	Total gained % points	Rank
**GROUP A (RFC = 0.9957–0.9009)**		
*Cymbopogon jwarancusa subsp*. *jwarancusa*	88.2	1st
*Typha elephantina*	87.3	2nd
*Cynodon dactylon*	87.1	3rd
*Cenchrus ciliaris*	85.1	4th
*Cyperus alopecuroides *	84	5th
**GROUP B (RFC = 0.8879–0.8103)**		
*Cenchrus pennisetiformis*	72.5	1st
*Lasiurus sindicus*	63.5	2nd
*Saccharum spontaneum*	62.4	3rd
*Tragus roxburghii*	60.9	4th
**GROUP C (RFC = 0.7974–0.6940)**		
*Enneapogon persicus *	77.9	1st
*Eragrostis atrovirens*	76.8	2nd
*Eragrostis pilosa*	72.1	3rd
*Phalaris minor*	70.1	4th
*Phragmites karka*	61.1	5th
**GROUP D (RFC = 0.6897–0.6121)**		
*Brachiaria ovalis*	72.1	1st
*Dichanthium annulatum*	60.3	2nd
*Aristida adscensionis*	59.9	3rd
*Bromus sericeus*	58.7	4th
*Celotia argentea*	55.9	5th
**GROUP E (RFC = 0.6034–0.6)**		
*Bromus pectinatus *	92.8	1st
*Fimbristylis quinquangularis*	90.5	2nd
*Poa annua*	85.2	3rd
*Stipagrostis plumosa*	76.9	4th
**GROUP F (RFC = 0.5431–0.5086)**		
*Themeda anathera*	59.1	1st
*Cenchrus setigerus*	55.6	2nd
*Cyperus imbricatus *	54.9	3rd
*Digitaria ciliaris *	52.3	4th

The species included in Group A (high priority) are ecologically dominant and largely available in the area. Moreover, taxa included in this group have a good palatability and are also available during the dry season when other grazing resources are exhausted.

### Palatability of grasses and the method of feeding

**Fig 8:**
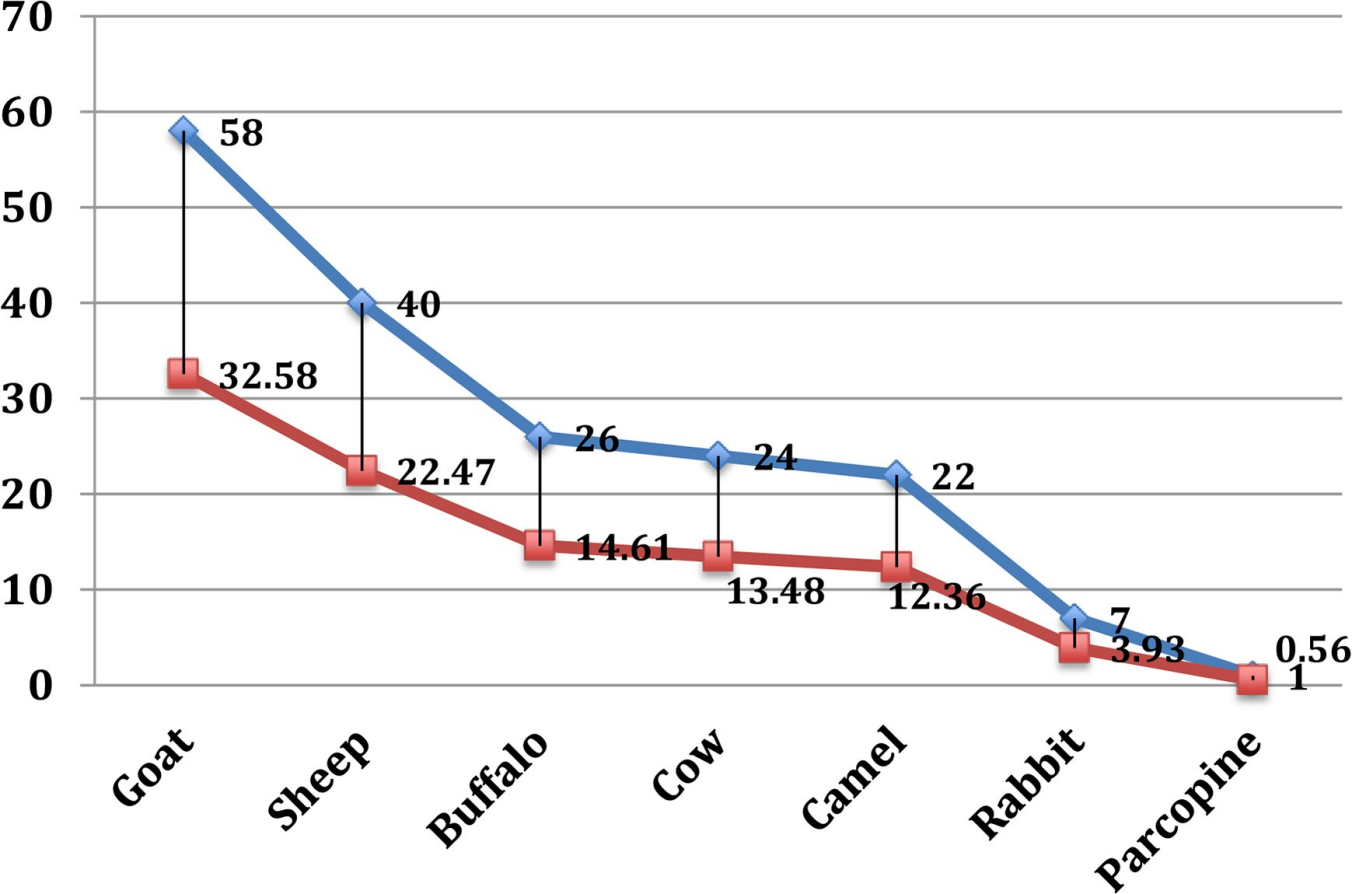
Grasses preference by animals.

Preferred palatable species are often leafy, with less stem, a low leaf table and leaves of low tensile strength [37,38]. Palatability analysis showed that 77% of the reported species are grazed in the study area (Table 7). In particular; grasses included in the group A of the priority ranking were consumed by all ruminants locally raised. Goats are the only animals to feed on every type of grass growing in Thal desert although palatability results show a preference for 58% of the reported species. 40% of the species represented the favorite fodder for sheep and 26% the favorite fodder for buffaloes. Camels are very selective animals and use only few specific grasses as fodder (Fig 8). Different parts showed to have different edibility: for example 42% of grass species were consumed as whole plant (e.g. *Cynodon dactylon*, *Eragrostis minor*, *Cenchrus ciliaris*, *Cenchrus pennisetiformis*, etc.) while 38% and 19% of them were consumed as aerial parts and as leaves, respectively. The reason why so many grasses are grazed as a whole is probably related to their small size and tender herbaceous texture (e.g. *Cynodon dactylon*, *Lasiurus sindicus*, *Phalaris minor*, *Cyperus rotundus*, *Eragrostis minor* etc. similar results shown in other literature [12, 13]. Due to the sandy nature of soils occurring in the study area these plants have shallow root systems and can easily be pulled out from the soil. Species growing in the form of dense patches are hard to be consumed as a whole and animals feed only on the aerial parts. Beliefs on livestock feeding habits are common in the area: for example, some local shepherds reported that putting the herd out to pasture in open fields improves their health and milk production. According to them freely grazing animals are able to select the best grasses, avoiding the toxic or less nutritious ones. They justify this belief by comparing milk production of freely grazing animals with forage-fed cattle and also by saying that during dry season, when free grazing is not possible, there is a considerable reduction in animal health and milk production [38, 39].

**Table 7:** Frequency analysis for palatability, parts used for eating and feeding methods and relative abundance of fodder grasses.

Studied parameters	Frequency	Valid percent	Cumulative percent
Co, Bu, Sh, Go, Ra	1	1.64	1.64
Co, Bu, Sh, Go	6	9.84	11.48
Co, Bu, Sh, Go, Ra	4	6.56	18.03
Go, Sh, Co	3	4.92	22.95
Go, Sh	20	32.79	55.74
Go, Sh, Co, Cm	1	1.64	57.38
Co, Bu, Sh, Go, Cm	11	18.03	75.41
Bu, Sh, Go	1	1.64	77.05
Co, Bu	3	4.92	81.97
Go	9	14.75	96.72
Sh	2	3.28	100
**Total**	**61**	**100**	
Whole plant	42	68.85	68.85
Leaves	4	6.56	75.41
Juvenile	2	3.28	78.69
Aerial, whole plant at Juvenile	1	1.64	80.33
Aerial, Juvenile	2	3.28	83.61
Aerial and leaves	2	3.28	86.89
Aerial	8	13.11	100.00
**Total**	**61**	**100**	
Fo	31	50.82	50.82
Fo,For, Mf	7	11.48	62.30
Fo, Mf	21	34.43	96.72
Fo,Mf	2	3.28	100.00
**Total**	**61**	**100**	
Abundant	30	49.18	49.18
Common	13	21.31	70.49
Frequent	9	14.75	85.25
Occasional	5	8.20	93.44
Rare	4	6.56	100
**Total**	**61**	**100**	

Key: Co (Cow), Bu (Buffalo), Sh (Sheep), Go (Goat), Ra (Rabbit), Cm (Camel), Fo (Fodder), For (Forage), Mf (Mix Fodder)

### Role of the fodder species on milk production

**Fig 9:**
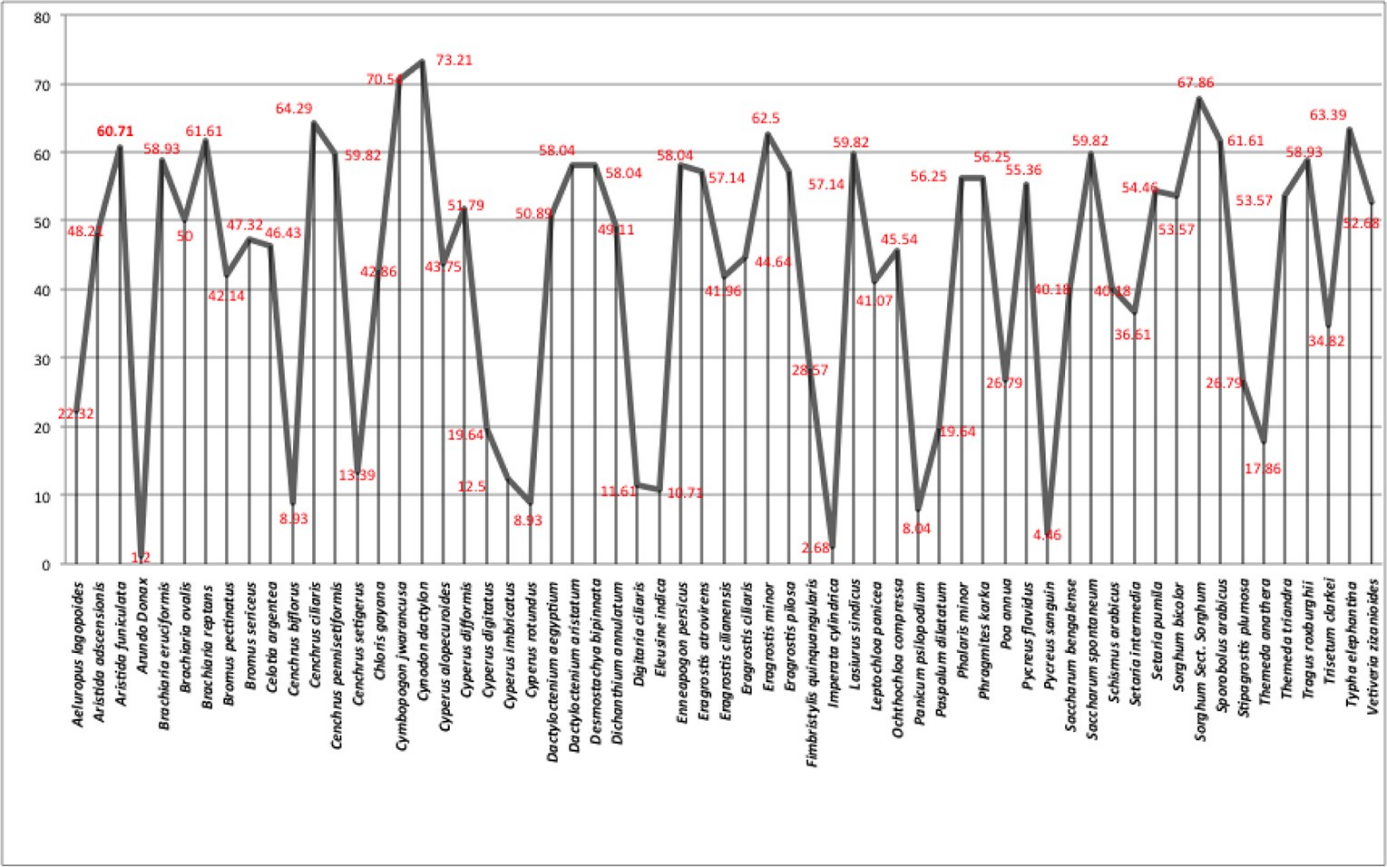
Frequency of milk producing species according to participants ranking.

Ten out of the 80 interviewed shepherds (based on the respondent knowledge) were randomly sampled to examine in detail the role of fodder species on the milk production. We focused our attention on the shepherds because, during the interviews, they showed a deeper knowledge about the plant species influencing quantity and quality of milk. According to them, *Cynodon dactylon* was the best species for milk production (6.46 SI, 0.6460 CS) followed by *Cymbopogon jwarancusa* (5.133 SI, 0.5133 CS). *Cymbopogon jwarancusa* was also reported to give a peculiar aroma, increasing the milk’s value. *Sorghum* sp. was the third most salient species (5.121 SI, 0.5121 CS) (Table 8). This findings were confirmed when we extended our analysis to all the participants. According to the results of the ANTHROPAC frequency analysis, ranking the plants in the order of their citation frequency (Fig 9), *Cynodon dactylon* had 73.21% frequency of milk production, following by *Cymbopogon jwarancusa* (70.54%) and *Sorghum* sp. (67.86%).

**Table 8:** Results of ANTHROPAC analysis of overall salience index of milk for producing species.

**S. No.**	Botanical name	Inverted Rank/Total Listed = Smith,s Salience Index										Illness Σ	Composite Salience Σ/n (n = 10)
		(SS1)	(SS2)	(SS3)	(SS4)	(SS5)	(SS6)	(SS7)	(SS8)	(SS9)	(SS10)		
1	*Cynodon dactylon*	1		0.96		0.9	0.883		1	0.867	0.85	6.46	0.6460
2	*Cymbopogon jwarancusa*	0.933	1		1	0.15		0.3	0.883		0.867	5.133	0.5133
3	*Sorghum* Sect. *Sorghum*	0.96	1.00		0.82	0.67	0.4	0.4	0.21	0.34	0.321	5.121	0.5121
4	*Cenchrus ciliaris*	0.933		0.75	0.933	0.42	0.51	0.51		0.4	0.15	4.606	0.4606
5	*Typha elephantina*	0.933	0.96	0.321	0.05	0.75	0.3	0.152	0.04	0.321	0.058	3.885	0.3885
6	*Eragrostis minor*	0.867		0.34		0.867	0.82		0.017	0.82		3.731	0.3731
7	*Brachiaria reptans*	0.62	0.72	0.321	0.05	0.75	0.3	0.152	0.04	0.321	0.058	3.332	0.3332
8	*Sporobolus arabicus*	0.85	0.767		0.783		0.017		0.532		0.33	3.279	0.3279
9	*Aristida funiculata*		0.82	0.67			0.083	0.768		0.083	0.833	3.257	0.3257
10	*Cenchrus pennisetiformis*	0.833		0.767	0.096	0.073		0.767	0.096	0.073	0.767	3.472	0.3472
11	*Lasiurus sindicus*	0.076	0.017	0.8	0.0764	0.0432	0.054	0.098	0.76	0.87	0.0973	2.8919	0.2892
12	*Saccharum spontaneum*	0.767	0.82	0.67		0.017			0.3	0.152	0.04	2.766	0.2766
13	*Tragus roxburghii*	0.021	0.02	0.031	0.768	0.8	0.0764	0.017	0.767	0.096	0.017	2.6154	0.2615
14	*Brachiaria eruciformis*	0.769	0.767	0.096	0.073	0.083	0.098	0.063	0.65			2.599	0.2599
15	*Dactyloctenium aristatum*		0.017	0.767	0.096	0.073	0.57	0.767	0.096	0.017		2.403	0.2403
16	*Desmostachya bipinnata*	0.733		0.82	0.67		0.017			0.083		2.323	0.2323
17	*Enneapogon persicus *			0.734	0.083	0.15	0.07	0.0631	0.023	0.421	0.51	2.0541	0.2054
18	*Eragrostis atrovirens*	0.735	0.03	0.042	0.15	0.768	0.12	0.032	0.027	0.053	0.0564	2.0154	0.2015
19	*Eragrostis pilosa*	0.0432	0.054	0.15	0.076	0.217	0.717	0.021	0.52	0.031	0.096	1.9252	0.1925
20	*Phalaris minor*	0.8	0.0764	0.017		0.083	0.033	0.05	0.032	0.083	0.7	1.8744	0.1874
21	*Phragmites karka*	0.701	0.01	0.023	0.74	0.15	0.07	0.0631	0.023		0.032	1.8161	0.1816
22	*Pycreus flavidus*			0.683	0.096	0.01	0.023	0.23	0.7	0.032	0.027	1.805	0.1805
23	*Setaria pumila *	0.65		0.15	0.07	0.0631		0.7	0.07	0.0631	0.023	1.7892	0.1789
24	*Sorghum bicolor*	0.0764	0.0432	0.054	0.651	0.217	0.15	0.07	0.0631	0.017	0.437	1.7787	0.1779
25	*Themeda triandra*					0.68	0.076	0.23	0.117	0.021	0.652	1.776	0.1776
26	*Vetiveria zizanioides*			0.617		0.15	0.07	0.0631	0.7			1.6001	0.1600
27	*Cyperus difformis*	0.6			0.0432	0.054	0.15	0.076	0.217		0.43	1.5702	0.1570
28	*Dactyloctenium aegyptium*	0.0432	0.054	0.651	0.567		0.0764	0.017		0.027	0.07	1.5056	0.1506
29	*Brachiaria ovalis*	0.13	0.51		0.0764	0.0432	0.054	0.651		0.017		1.4816	0.1482
30	*Dichanthium annulatum*	0.132	0.242	0.517	0.15	0.076	0.017	0.15	0.07	0.0631	0.023	1.4401	0.1440
31	*Aristida adscensionis*	0.5	0.142		0.251	0.217		0.01	0.023	0.23	0.017	1.394	0.1394
32	*Bromus sericeus*		0.054		0.45			0.083	0.054	0.051	0.567	1.259	0.1259
33	*Celotia argentea*	0.433				0.0432	0.054	0.051	0.567		0.0764	1.2246	0.1225
34	*Ochthochloa compressa*	0.076	0.23	0.4	0.071		0.083	0.0764		0.0432	0.054	1.0336	0.1034
35	*Eragrostis ciliaris*	0.367		0.076	0.23	0.14	0.07		0.15	0.083		1.116	0.1116
36	*Cyperus alopecuroides *		0.251		0.333	0.0432	0.054	0.051	0.05	0.025	0.142	0.9492	0.0949
37	*Chloris gayana *	0.3	0.142	0.071		0.01	0.023	0.23	0.017	0.0631	0.023	0.8831	0.0883
38	*Eragrostis cilianensis*	0.051		0.083	0.076	0.023	0.4	0.071	0.051	0.05		0.805	0.0805
39	*Leptochloa panicea*	0.284	0.026	0.0432	0.054		0.14	0.07	0.083	0.054	0.033	0.7872	0.0787
40	*Saccharum bengalense*	0.02	0.0710	0.055	0.046	0.0532	0.064	0.25	0.01	0.083	0.054	0.7142	0.0714
41	*Schismus arabicus *	0.2			0.061		0.05	0.083	0.076	0.023	0.22	0.713	0.0713
42	*Setaria intermedia *	0.055	0.067		0.183		0.0132	0.211	0.071	0.051	0.05	0.7012	0.0701
43	*Trisetum clarkei*	0.167		0.233	0.0432	0.054	0.026	0.0432	0.054	0.011	0.04	0.6714	0.0671
44	*Bromus pectinatus *		0.14	0.15			0.017	0.117	0.15	0.076	0.017	0.667	0.0667
45	*Fimbristylis quinquangularis *	0.151		0.0432		0.046	0.0532	0.064	0.25	0.017		0.6244	0.0624
46	*Poa annua*	0.071	0.051	0.05		0.017	0.233	0.0432	0.152			0.6172	0.0617
47	*Stipagrostis plumosa*	0.117		0.017	0.071	0.051	0.05	0.017		0.233	0.0432	0.5992	0.0599
48	*Aeluropus lagopoides*	0.055	0.161	0.0432	0.054	0.011	0.033	0.055	0.026	0.0432	0.054	0.5354	0.0535
49	*Cyperus digitatus *	0.1	0.023	0.046	0.0532	0.064		0.05	0.0710	0.055	0.046	0.5082	0.0508
50	*Paspalum dilatatum* Poir.	0.032	0.042	0.217	0.102		0.046		0.046	0.017		0.502	0.0502
51	*Themeda anathera*	0.0432	0.054	0.011	0.033	0.25			0.083			0.4742	0.0474
52	*Cenchrus setigerus*	0.0432	0.054	0.017	0.0432	0.054	0.071	0.051	0.05		0.084	0.4674	0.0467
53	*Cyperus imbricatus *		0.051		0.067		0.071	0.051		0.0432	0.152	0.4352	0.0435
54	*Digitaria ciliaris*						0.067	0.233	0.0432	0.054	0.026	0.4232	0.0423
55	*Eleusine indica*	0.05	0.0432	0.233			0.026		0.054			0.4062	0.0406
56	*Cyperus rotundus*	0.01		0.05	0.017	0.071	0.011	0.033	0.055	0.0710	0.055	0.373	0.0373
57	*Cenchrus biflorus*	0.033	0.017	0.0432	0.054	0.011	0.054	0.064	0.0432	0.017	0.011	0.3474	0.0347
58	*Panicum psilopodium *	0.033	0.017	0.071	0.051	0.05		0.033	0.033	0.033		0.321	0.0321
59	*Pycreus sanguin *	0.011	0.031	0.055	0.0110	0.023	0.011	0.033	0.055	0.0710	0.017	0.318	0.0318
60	*Imperata cylindrica*	0.017		0.033		0.011		0.046	0.0532	0.064	0.055	0.2792	0.0279
61	*Arundo Donax*	0.018			0.071	0.051	0.05		0.042	0.017		0.249	0.0249

### Relative abundance and seasonal availability

**Fig 10:**
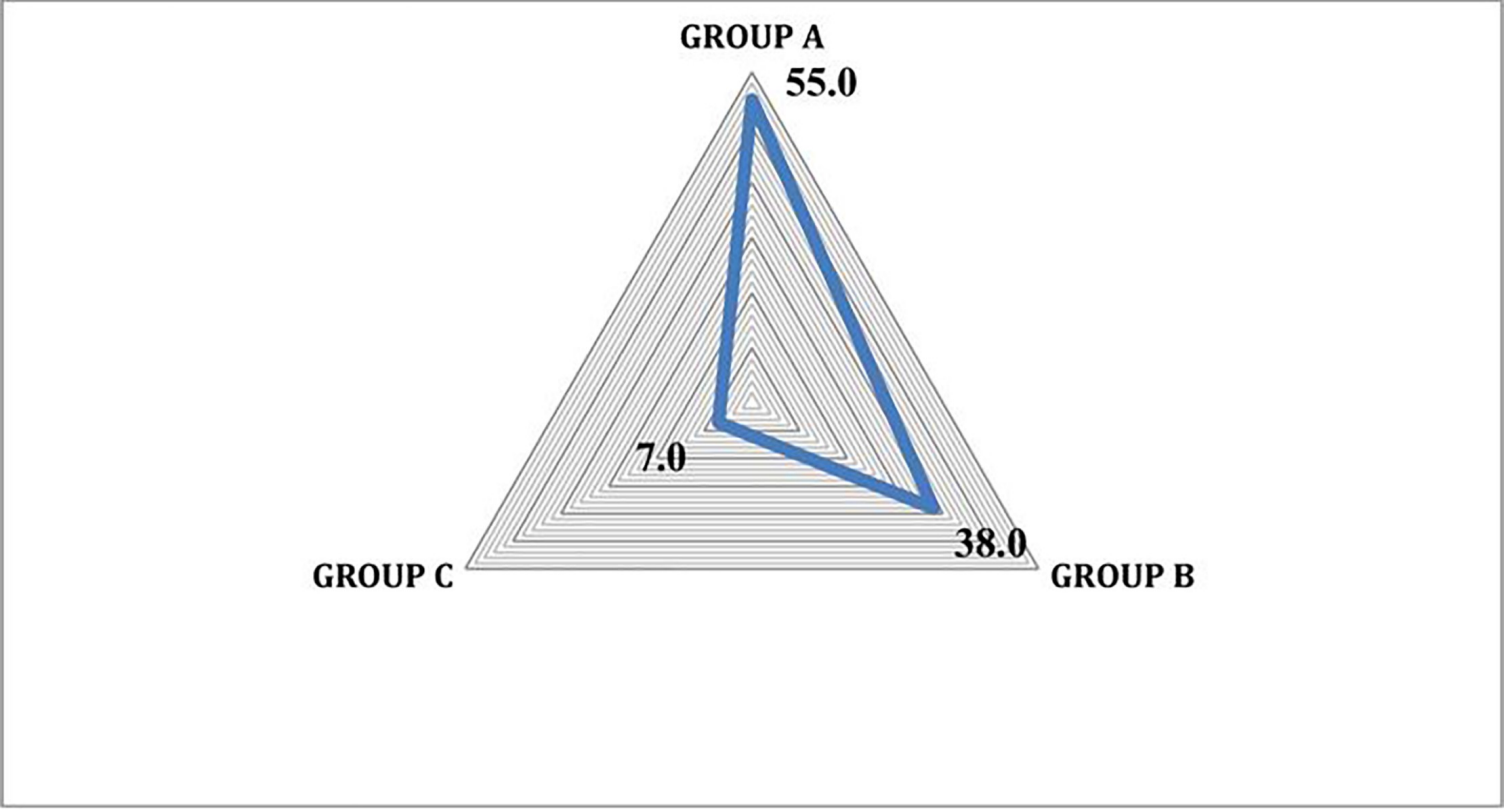
Percentage of species in each group.

**Fig 11:**
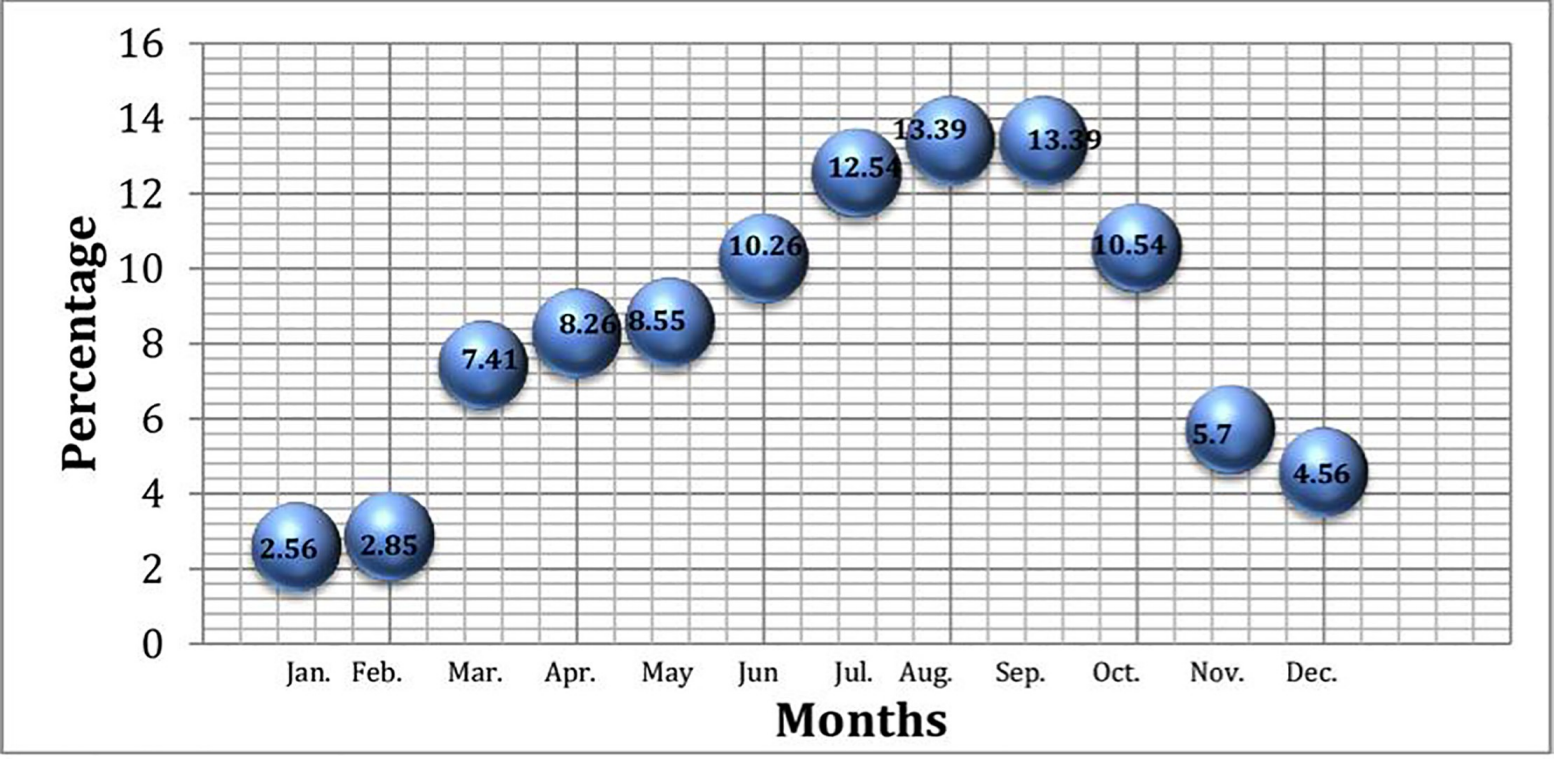
Availability of grasses in the study area.

Relative abundance analysis showed that most of the cited species (55%) were abundantly present in the study area and most of them belonged to priority Group A (Fig 10). 13.39% of the species were available in August and in October while 12.54% were available in July. In Pakistan, July, August and October are months characterized by monsoon rains fostering the grass biomass development (Fig 11).

### People use livestock for improving their economic life

**Fig 12:**
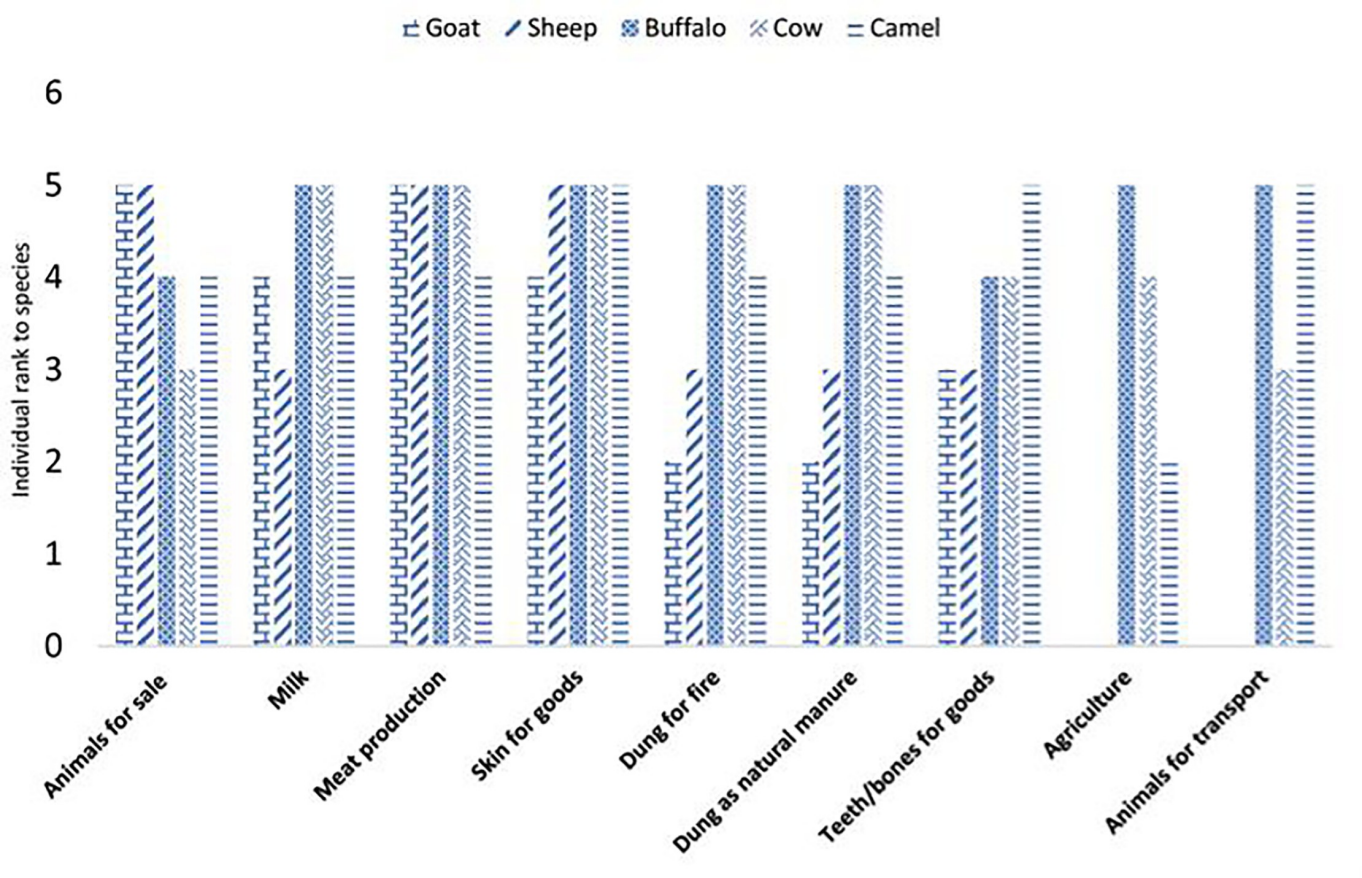
People use livestock for improving their economic life in Thal Desert.

Livestock production makes the main contribution to agriculture value-added services in the study area. Ten local participants were asked to rank animals from one to five on the basis of their economic value. Milk production is the major income source for people living in the Thal desert; for milk production, to cows and buffaloes are raised more frequently than camels or goats (Fig 12). Goats, sheep, buffaloes and cows are also raised for meat production. During religious celebrations (such as pilgrimages and Eid ul Azha) shepherds and farmers take livestock to the local market for sale and this is another major income source as also shown in [40]. Skins from sheep, buffaloes, cows and camels are also sold for making leather goods; teeth and bones are used for making different objects (e.g. buttons, jewelry and decoration pieces) (Fig 12). Dung of buffaloes and cows is dried and used as fuel or, fresh, as a natural fertilizer to improve the soil fertility. Ox, buffaloes and sometimes camels are used for ploughing. Camels are commonly used for transportation in desert areas.

### Indigenous uses and ethno-veterinary uses of grasses

Eighteen of the 61 reported species were locally used in ethno-veterinary practice. *Cymbopogon jwarancusa* was the most cited veterinary grass (48) and was reported to heal infertility and skin diseases in ruminants (Table 9). Other species (*Cenchrus* spp., *Arundo donax*, *Desmostachya bipinnata*, *Dichanthium annulatum*, *Digitaria ciliaris*, *Eleusine indica*, *Eragrostis* spp., *Saccharum spontaneum*) were frequently reported as remedies to treat urinary and digestive diseases in livestock. Similar results are shown in different studies [12, 41, 42]. Urinary and digestive diseases were the most frequently reported disorders; this finding is probably due to the sandy nature of the soil, causing the accumulation of sand-laden feed material in the digestive apparatus and urinary tract of livestock. Fic analysis showed in Table 5 that according to the Participents *Cenchrus ciliaris*, *Cenchrus biflorus*, *Desmostachya bipinnata*, *Cyperus rotundus*, *Cynodon dactylon*, *Digitaria ciliaris* and *Saccharum spontaneum* are important for veterinary uses.

**Table 9:** Grasses use in ethno-veterinary and ethnobotanical uses of grasses.

S. No.	Botanical name	Ethnobotanical Uses	Ethno veterinary uses
** 1**	*Aeluropus lagopoides* (L.) *Thwaites*	Fuel	---
2	*Aristida adscensionis* L	---	Controls itching
3	*Arundo Donax L*.	---	Gastrointestinal
4	*Arundo Donax L*.	Fencing, inkpot pen, hollow stem for announcement	---
5	*Brachiaria ovalis* Stapf	Fuel	---
6	*Bromus pectinatus* Thunb.	Fuel	---
7	*Bromus sericeus* Drobov	Fuel	---
8	*Cenchrus biflorus* Roxb.	---	Diuretic
9	*Cenchrus ciliaris* L.	Fuel	Diuretic
10	*Cenchrus pennisetiformis* Hochst. & Steud.	Fuel	---
11	*Cenchrus setigerus* Vahl	Fuel	Diuretic
12	*Cymbopogon jwarancusa subsp*. *jwarancusa* (Jones) Schult.	Fumigant for measles, matrices (Chatai) for typhoid, root extract for typhus fever and cough, Seeds for chicken pox, roof thatching, roots khass for washing domestic pots/utensils	Fumigant for skin diseases, fragrance in milk, Diuretic and improve fertility in bull
13	*Cynodon dactylon* (L.) Pers.	Remove pimples, feet burning sensation, fever	Paste of leaves controls dysentery and anti-inflammatory to wounded areas of animal’s body
14	*Cyperus digitatus *Roxb.	Fuel	**---**
15	*Cyperus rotundus* L.	Fuel	Antidiarrheal and gur function stabilizer
16	*Dactyloctenium aegyptium* (L.) Willd.	---	Used to reduce after birth abdominal pains
17	*Desmostachya bipinnata* (L.) Stapf.	Broom making, Fuel	Digestive disorders, Dysentery
18	*Dichanthium annulatum* (Forssk.) Stapf	Fuel	Digestive disorders
19	*Digitaria ciliaris* (Retz.) Koel	Fuel	
20	*Eleusine indica* (L.) Gaertn.	---	Cure digestive disorders
21	*Eragrostis minor* Host	---	Digestive disorders
22	*Eragrostis pilosa (Linn*.*) P*. *Beauv*.	---	Help to cure contusion
23	*Imperata cylindrica* (L.) Raeuschel.	---	Fumigant for Piles
24	*Leptochloa panicea* (Retz.) Ohwi	Fuel	---
25	*Phragmites karka* (Retz.) Trin. ex Steud.	Writing pen (Qalam) trunk, thatching of roof, and fuel source, shoes making	---
26	*Pycreus flavidus* (Retz.) T. Koyama	Fuel	---
27	*Saccharum bengalense* Retz.	Culms used for making matrices, chairs (Morrhe), hand fan, cages (Pinjra), brooms (Jhaaru), etc. Leaves used for making matrices (Chatai). Leaf sheaths beaten to make strong ropes (Rassi)	Leaves used to treat oral problems of ruminants
28	*Saccharum spontaneum* L.	Leaves Decoction for stoppage of urination (Micturition),fuel, culm used for making cages, roof thatching (Patalan) and ornamental goods. Leaves woven to make matrices	Root help to relieve in inflammation and urinary problems
29	*Sorghum bicolor* (Linn.) Moench.	Fuel	Wounds, fever, anemia and constipation
30	*Sorghum* Sect. *Sorghum* Subsect. *Arundinacea Moench*.	Fuel	---
31	*Sporobolus arabicus* Boiss.	Fuel	---
32	*Tragus roxburghii* Panigrahi	Fuel	---
33	*Typha elephantina* Roxb.	Fuel, roof thatching, ropes, matrices, inflorecese medicinally importance and shoes making	---

## Conclusion

The present study provides an inventory of plant species, plant parts and diversity in palatability and feeding behavior. The data analysis highlighted the possible motives behind the greater acceptability ratio of high priority fodder grasses: i.e. diversity in their palatability for major ruminant species, availability in the study area, and versatility of feeding methods. This study is not only significant for the conservation of ethnobotanical knowledge but may also help in facilitating sustainable feeding for ruminants. Subsequently, the information may play a major role in improving the livelihood of smallholder farmers. A blend of traditional and scientific knowledge is required to produce a worthwhile criterion for selecting fodder grasses. If some of the grasses show promising nutritional and pharmacological value, then necessary steps to conserve the area and the species should be taken. This will help to boost up the economy of the country.

## References

[1] Ghazali A. Analyzing the relationship between foreign direct investment domestic investment and economic growth for Pakistan. International Research Journal of Finance and Economics. 2010;47(1):123–31.

[2] Farooq U, Ahmad M, Saeed I. Enhancing livestock productivity in the desert ecologies of Pakistan: setting the development priorities. Pakistan Development Review. 2009;48(4):795–823.

[3] Hussain F, Durrani MJ. Seasonal availability, palatability and animal preferences of forage plants in Harboi arid range land, Kalat, Pakistan. Pakistan Journal of Botany (Pakistan). 2009.

[4] Okoli I, Ebere C, Uchegbu M, Udah C, Ibeawuchi I. A survey of the diversity of plants utilized for small ruminant feeding in south-eastern Nigeria. Agriculture, ecosystems & environment. 2003;96(1–3):147–54.

[5] Bahru T, Asfaw Z, Demissew S. Ethnobotanical study of forage/fodder plant species in and around the semi-arid Awash National Park, Ethiopia. Journal of forestry research. 2014;25(2):445–54.

[6] Bruschi P, Urso V, Solazzo D, Tonini M, Signorini MA. Traditional knowledge on ethno-veterinary and fodder plants in South Angola: an ethnobotanic field survey in Mopane woodlands in Bibala, Namibe province. Journal of Agriculture and Environment for International Development (JAEID). 2017;111(1):105–21.

[7] Nunes AT, Lucena RFP, dos Santos MVF, Albuquerque UP. Local knowledge about fodder plants in the semi-arid region of Northeastern Brazil. Journal of ethnobiology and ethnomedicine. 2015;11(1):12. 25972095 10.1186/1746-4269-11-12 PMC4429921

[8] Singh V, Gaur R, Bohra B. A survey of fodder plants in mid-altitude Himalayan rangelands of Uttarakhand, India. Journal of Mountain Science. 2008;5(3):265–78.

[9] Nautiyal S. Interactions Between Humans and Ecosystems in Himalayas of India and Its Socioeconomic and Ecological Consequences: An Ecological Modelling Approach. Ecosystem Functions and Management: Springer; 2017 p. 39–57.

[10] Abdullah M, Rafay M, Hussain T, Ahmad H, Tahir U, Rasheed F, et al NUTRITIVE POTENTIAL AND PALATABILITY PREFERENCE OF BROWSE FOLIAGE BY LIVESTOCK IN ARID RANGELANDS OF CHOLISTAN DESERT (PAKISTAN). JAPS, Journal of Animal and Plant Sciences. 2017;27(5):1656–64.

[11] Zareen A, Khan Z, Ajaib M. Ethnobotanical evaluation of the shrubs of Central Punjab, Pakistan. Biologia (Pakistan). 2013;59(1):139–47.

[12] Harun N, Chaudhry AS, Shaheen S, Ullah K, Khan F. Ethnobotanical studies of fodder grass resources for ruminant animals, based on the traditional knowledge of indigenous communities in Central Punjab Pakistan. Journal of ethnobiology and ethnomedicine. 2017;13(1):56 10.1186/s13002-017-0184-5 28978348 PMC5628460

[13] Geng Y, Hu G, Ranjitkar S, Wang Y, Bu D, Pei S, et al Prioritizing fodder species based on traditional knowledge: a case study of mithun (Bos frontalis) in Dulongjiang area, Yunnan Province, Southwest China. Journal of ethnobiology and ethnomedicine. 2017;13(1):24 10.1186/s13002-017-0153-z 28472968 PMC5418811

[14] Shaheen H, Qureshi R, Akram A, Gulfraz M, Potter D. A preliminary floristic checklist of Thal Desert Punjab, Pakistan. Pak J Bot. 2014;46(1):13–8.

[15] Khalil AT, Shinwari ZK, Qaiser M, Marwat KB. Phyto-therapeutic claims about euphorbeaceous plants belonging to Pakistan; an ethnomedicinal review. Pak J Bot. 2014;46(3):1137–44.

[16] Abbasi AM, Khan SM, Ahmad M, Khan MA, Quave CL, Pieroni A. Botanical ethnoveterinary therapies in three districts of the Lesser Himalayas of Pakistan. Journal of ethnobiology and ethnomedicine. 2013;9 10.1186/1746-4269-9-9 24359615 PMC3904763

[17] Khan M, Khan MA, Mujtaba G, Hussain M. Ethnobotanical study about medicinal plants of Poonch valley Azad Kashmir. J animal plant Sci. 2012;22:493–500.

[18] Hussain I. Profile of Livestock Production in Thal Desert of Pakistan. International Journal of Academic Research in Business and Social Sciences. 2017;7(3):480–94.

[19] Faraz A, Waheed A, Ishaq H, Mirza R. Rural Development by Livestock Extension Education in Southern Punjab. J Fisheries Livest Prod. 2019;7(287):2.

[20] Waters J. Snowball sampling: a cautionary tale involving a study of older drug users. International Journal of Social Research Methodology. 2015;18(4):367–80.

[21] Ahmad Z, Zamhuri KF, Yaacob A, Siong CH, Selvarajah M, Ismail A, et al In vitro anti-diabetic activities and chemical analysis of polypeptide-k and oil isolated from seeds of Momordica charantia (bitter gourd). Molecules. 2012;17(8):9631–40. 10.3390/molecules17089631 22885359 PMC6268611

[22] ISo E. ISE Code of Ethics (with 2008 additions). International Society of Ethnobiology; 2006.

[23] Zubair M, Khan S, Hussain SB, Haq AU, Jamil A. Ethnobotanical Study Of Pakistan’s Southern Punjab Tehsil Of Dunyapur. International Journal of Multidisciplinary Research and Studies. 2019;2(09):40–52.

[24] Shaheen H, Qureshi R, Qaseem MF, Amjad MS, Bruschi P. The cultural importance of indices: A comparative analysis based on the useful wild plants of Noorpur Thal Punjab, Pakistan. European Journal of Integrative Medicine. 2017;12:27–34.

[25] Shaheen H, Qureshi R, Akram A, Gulfraz M. Some important medicinal flora of Noorpur Thal, Khushab, Pakistan. Archives Des Sciences. 2012;65(2):57–73.

[26] Shaheen H, Qaseem MF, Amjad MS, Bruschi P. Exploration of ethno-medicinal knowledge among rural communities of Pearl Valley; Rawalakot, District Poonch Azad Jammu and Kashmir. PloS one. 2017;12(9):e0183956 10.1371/journal.pone.0183956 28886077 PMC5590857

[27] Nadaf M, Joharchi M, Amiri MS. Ethnomedicinal uses of plants for the treatment of nervous disorders at the herbal markets of Bojnord, North Khorasan Province, Iran. Avicenna journal of phytomedicine. 2019;9(2):153 30984580 PMC6448544

[28] Qaseem M, Qureshi R, Amjad M, Ahmed W, Masood A, Shaheen H. ETHNO-BOTANICAL EVALUATION OF INDIGENOUS FLORA FROM THE COMMUNITIES OF RAJH MEHAL AND GOI UNION COUNCILS OF DISTRICT KOTLI, AZAD JAMMU KASHMIR PAKISTAN. APPLIED ECOLOGY AND ENVIRONMENTAL RESEARCH. 2019;17(2):2799–829.

[29] Nankaya J, Nampushi J, Petenya S, Balslev H. Ethnomedicinal plants of the Loita Maasai of Kenya. Environment, Development and Sustainability. 2019:1–21.

[30] Tounekti T, Mahdhi M, Khemira H. Ethnobotanical Study of Indigenous Medicinal Plants of Jazan Region, Saudi Arabia. Evidence-Based Complementary and Alternative Medicine. 2019;2019. 10.1155/2019/3190670 PMC6582903 31275409

[31] Heinrich M, Ankli A, Frei B, Weimann C, Sticher O. Medicinal plants in Mexico: Healers' consensus and cultural importance. Social Science & Medicine. 1998;47(11):1859–71. 9877354 10.1016/s0277-9536(98)00181-6

[32] Torres-Avilez W, Medeiros PMd, Albuquerque UP. Effect of gender on the knowledge of medicinal plants: systematic review and meta-analysis. Evidence-Based Complementary and Alternative Medicine. 2016;2016. 10.1155/2016/6592363 PMC5067321 27795730

[33] Khan AA, Khan K. Womenâ€^™^ s Role in Livestock Economy of Cholistan Desert, Pakistan. Global Journal of Human-Social Science Research. 2015.

[34] Voeks RA, Leony A. Forgetting the forest: assessing medicinal plant erosion in eastern Brazil. Economic Botany. 2004;58(sp1):S294–S306.

[35] Upadhyay B, Roy S, Kumar A. Traditional uses of medicinal plants among the rural communities of Churu district in the Thar Desert, India. Journal of ethnopharmacology. 2007;113(3):387–99. 10.1016/j.jep.2007.06.010 17714898

[36] de Melo JG, Santos AG, de Amorim ELC, Nascimento SCd, de Albuquerque UP. Medicinal plants used as antitumor agents in Brazil: an ethnobotanical approach. Evidence-based complementary and alternative medicine. 2011;2011. 10.1155/2011/365359 PMC3082129 21528006

[37] de Lucena RFP, de Medeiros PM, de Lima Araújo E, Alves AGC, de Albuquerque UP. The ecological apparency hypothesis and the importance of useful plants in rural communities from Northeastern Brazil: An assessment based on use value. Journal of Environmental Management. 2012;96(1):106–15. 10.1016/j.jenvman.2011.09.001 22208403

[38] Meissner H. Recent research on forage utilization by ruminant livestock in South Africa. Animal Feed Science and Technology. 1997;69(1–3):103–19.

[39] Provenza FD, Villalba JJ, Dziba L, Atwood SB, Banner RE. Linking herbivore experience, varied diets, and plant biochemical diversity. Small ruminant research. 2003;49(3):257–74.

[40] Haleem S. Pakistan-Culture Smart!: The Essential Guide to Customs & Culture: Bravo Limited; 2013 10.1007/s10549-017-4496-x

[41] Abbasi AM, Khan MA, Shah MH, Shah MM, Pervez A, Ahmad M. Ethnobotanical appraisal and cultural values of medicinally important wild edible vegetables of Lesser Himalayas-Pakistan. J Ethnobiol Ethnomed. 2013;9(1):66 Epub 2013/09/17. 10.1186/1746-4269-9-66 24034131 PMC3853161

[42] Moreki JC. Documentation of ethnoveterinary practices used in family poultry in Botswana. Vet World. 2013;6(1):18–21.

