# Correction: The fodder grass resources for ruminants: A indigenous treasure of local communities of Thal desert Punjab, Pakistan

**DOI:** 10.1371/journal.pone.0293099

**Published:** 2023-10-12

**Authors:** Humaira Shaheen, Rahmatuallah Qureshi, Mirza Faisal Qaseem, Piero Bruschi

There are errors in the Funding statement. The correct Funding statement is as follows: The study was funded by Chinese Academy of Sciences President’s International Fellowship Initiative (CAS PIFI: Grant No. 2019PB0105). The Higher Education Commission (HEC), Pakistan, also provided a travel grant for visit to USA to improve the data analyzing skills.[1]
